# Supplementary figures and images for: Genetic variation in the FMO and GSTO gene clusters impacts arsenic metabolism in humans
Source: PLoS Genet. 2025 Sep 2;21(9):e1011826. doi: 10.1371/journal.pgen.1011826 (PMC12404487; doi:10.1371/journal.pgen.1011826)

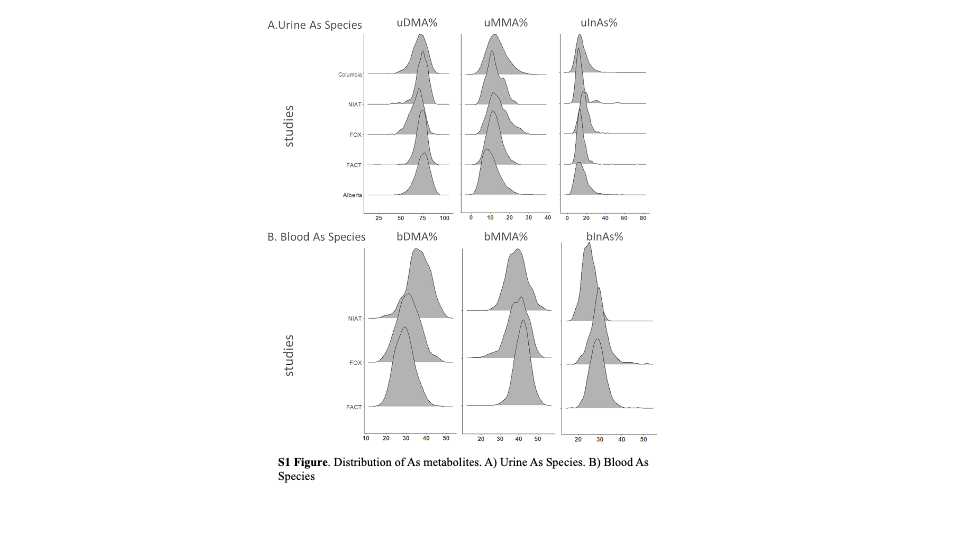

Supplement: S1 Fig — A) Urine As Species (Total n = 6,540), Columbia, n = 3, 687, NIAT, n = 163, FACT, n = 594, FOX, n = 341, Alberta, n = 1800. B) Blood As Species (Total n = 977), NIAT, n = 110, FOX, n = 273, FACT, 594. (TIFF) [file pgen.1011826.s001.tiff]

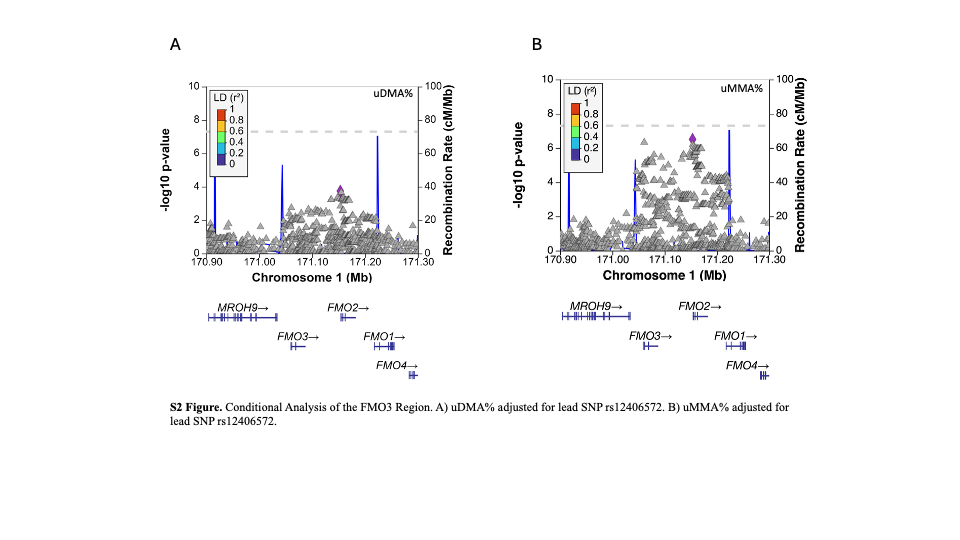

Supplement: S2 Fig — A) uDMA% results adjusted for lead SNP rs12406572. B) uMMA% results adjusted for lead SNP rs12406572. (TIFF) [file pgen.1011826.s002.tiff]

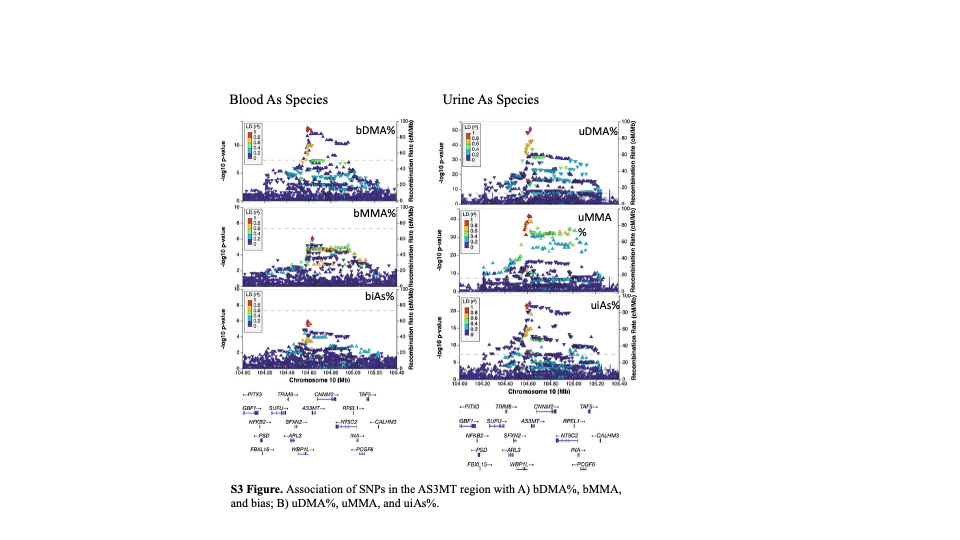

Supplement: S3 Fig — (TIFF) [file pgen.1011826.s003.tiff]

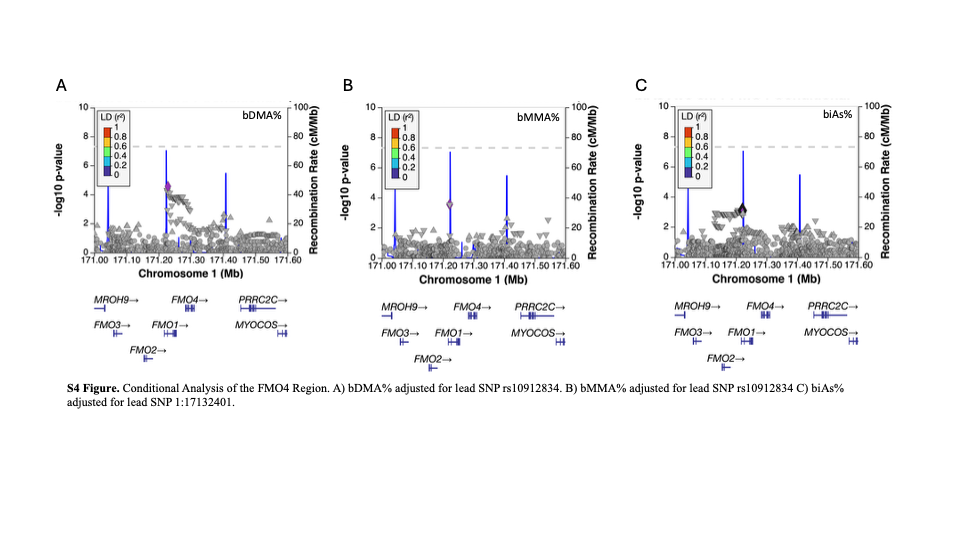

Supplement: S4 Fig — A) bDMA% adjusted for lead SNP rs10912834. B) bMMA% adjusted for lead SNP rs10912834 B) biAs% adjusted for lead SNP rs2011345. (TIFF) [file pgen.1011826.s004.tiff]

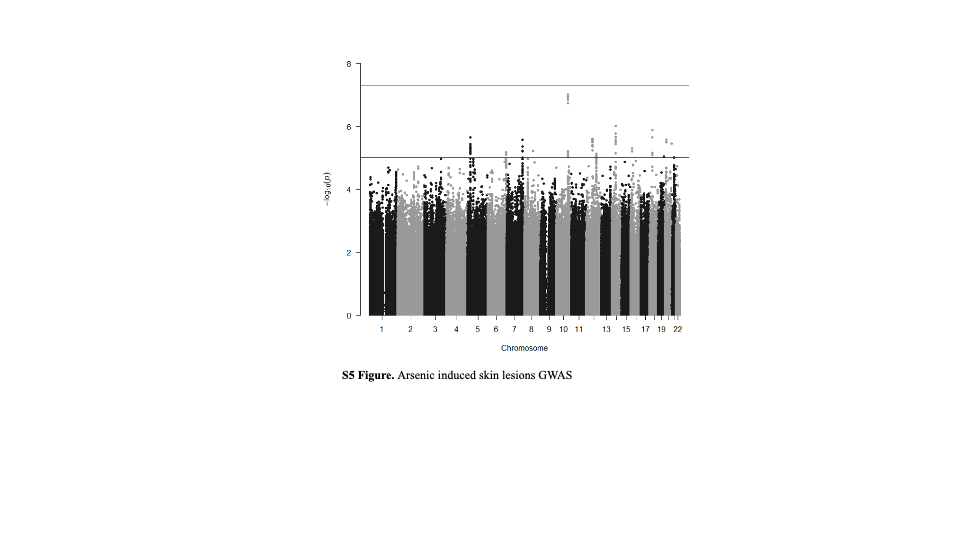

Supplement: S5 Fig — (TIFF) [file pgen.1011826.s005.tiff]
