# Supplementary material for: Genetic variation in the FMO and GSTO gene clusters impacts arsenic metabolism in humans
Source: PLoS Genet. 2025 Sep 2;21(9):e1011826. doi: 10.1371/journal.pgen.1011826 (PMC12404487; doi:10.1371/journal.pgen.1011826)
Supplement: Attachment — Submitted filename: Reviewer_response.docx [file pgen.1011826.s011.docx]

**Responses to reviewer comments**

We thank the reviewers for their insightful comments on our submission. These suggestions have helped improve the clarity and scope of our work. Our responses are below.

Reviewer #1: Review for “Genetic variation in the FMO and GSTO gene clusters impacts arsenic metabolism in humans”

Tamayo and colleagues performed GWA and colocalization analyses to test the association between arsenic species and common genetic variants. These analyses replicated previously observed relationships with arsenic and genetic variants in AS3MT and FTCD. In addition, novel variants were observed in FMO3, FMO4, and GSTO1. Colocalization analyses showed colocalization of these variants with several tissues in GTEx.

General Comments:
This study provides additional insight into potential mechanism of arsenic processing in the human body.

Methods and Materials:
1. As arsenic was measured at multiple timepoints, do you know if the concentration of arsenic is consistent across time points? Have you considered a longitudinal GWAS if not?

Answer:

**RESPONSE:** In the HEALS cohort, we have longitudinal data on arsenic levels in urine, but we do not have longitudinal data on arsenic species in urine. All arsenic species measurements were conducted using baseline samples. In this study, we are performing a GWAS on arsenic species, not on arsenic levels, thus, we cannot perform a longitudinal GWAS of arsenic species.

Regarding arsenic concentrations over time, past studies have shown that arsenic concentrations in individual wells remain within a relatively narrow range over time. Thus, we consider the arsenic levels in specific wells to be relatively stable across time points. However, exposure levels for HEALS participants have changed over time, as many participants switched wells from high arsenic wells to low arsenic wells after baseline, but these changes are not especially relevant to the manuscript under review.

Statistical Analysis
2. Page 6: Are you able to conduct a sensitivity analysis utilizing the environmental factors mentioned in the introduction? (Smoking, BMI, …) Either by controlling for these factors or performing a subgroup analysis.

**RESPONSE:** We conducted a sensitivity analysis by adding BMI and smoking to the model for the top FMO3 SNP associated with urinary As species (rs12406572), the top FMO4 SNP associated with blood As species (rs10912834) and the top GSTO1 SNP (**rs34521730)** associated with blood arsenic species. These associations remained largely unchanged after adjustments, with very small p-values persisting. This is expected as our SNPs of interest are uncorrelated with participant characteristics (other than AME variables). Below are the results of the sensitivity analysis:

### **Sensitivity Analysis Results**

**FMO3:rs12406572 (Urine)**

| **Outcome** | **Chr** | **SNP** | **β** | **p-value** |
| --- | --- | --- | --- | --- |
| Uine MMA% | 1 | 1:171084242:C:A | -1.4 | 1.43e-16 |
| Urine DMA% | 1 | 1:171084242:C:A | 1.6 | 2.89e-08 |

#### **FMO4: rs10912834 (Blood)**

| **Outcome** | **Chr** | **SNP** | **β** | **p-value** |
| --- | --- | --- | --- | --- |
| Blood MMA% | 1 | 1:171330006:A:C | 1.4 | 6.74e-12 |
| Blood DMA% | 1 | 1:171330006:A:C | -2.2 | 8.26e-24 |

#### **GSTO1: rs34521730 (Blood)**

| **Outcome** | **Chr** | **SNP** | **β** | **p-value** |
| --- | --- | --- | --- | --- |
| Blood MMA% | 10 | 10:106025030:A:T | -2.7 | 1.47e-07 |
| Blood DMA% | 10 | 10:106025030:A:T | 1.8 | 3.24e-13 |

In the manuscript, we now report the following in several locations: “*All associations were robust to adjustment for BMI and smoking status.”*

3. Is there concern for population substructure in these analyses? I don’t see the traditional PCA.

**RESPONSE:** Previous studies in HEALS have clearly demonstrated lack of population stratification in HEALS, based on principal components analysis (PCA). (e.g., Pierce BL, et al. *Genome-wide association study identifies chromosome 10q24.32 variants associated with arsenic metabolism and toxicity phenotypes in Bangladesh*, PLOS 2012). However, this prior work demonstrated the presence of cryptic relatedness in HEALS. To account for ancestry and relatedness, we used a mixed model approach (GCTA). This approach accounts for both cryptic relatedness and population stratification. Our QQ plots show minimal inflation, confirming adequate adjustment for population structure.

4. Have you considered a conditional GWAS to test the signals found?

**RESPONSE:** We conducted conditional analyses for each identified association signal, as described in the Results section (*GWAS of arsenic species measured in urine*). Specifically, for the newly identified **FMO3** and **FMO4** signals, evidence for secondary association signals are presented in *Supplemental Figure 2 and Supplementary Figure 4,* respectively.

Reviewer #2: Summary: This study identified the FMO and GSTO gene clusters as novel regions in which genetic variation influences arsenic species composition in humans. However, those newly identified metabolism-related variants did not show clear associations with arsenic-induced skin lesion risk.
Although the topic is interesting, there are several issues to be addressed to further improve the quality of the manuscript.

1. The statement in the conclusion “FMOs are involved in oxidation of xenobiotics, but have no known role in arsenic metabolism, while GSTO1 has a role in reducing arsenic species” is not very accurate. GSTO1 having a well-established role in catalyzing the reduction of arsenic species, but not in reducing arsenic species. The minor allele (T, MAF=10.3%) at lead SNP rs34521730 was associated decreased blood DMA%, increased blood MMA%, and increased blood iAs%, which means GSTO1 SNP is associated with reduced arsenic metabolism efficiency (AME)

**RESPONSE:** This statement has now been changed to the following to better acknowledge GSTO’s role in catalyzing the reduction of arsenic species in the conclusion of the abstract: “F*MOs are involved in the oxidation of xenobiotics but have no known direct role in arsenic metabolism, while GSTO1 has a well-established role in catalyzing the reduction of arsenic species*”

2. In the methodology section, it is necessary to provide a detailed introduction to the detection methods of arsenic species in different studies, as this is the most important factor in ensuring the stability and reliability of research results.

**RESPONSE:** We have expanded the text below to better describe detection of arsenic species in the different studies included in our manuscript:

Both urinary and blood arsenic species were distinguished using a method described by Reuter et al.^31^ This method entails using high-performance liquid chromatography (HPLC) separation of arsenobetaine, arsenocholine, As^V^, As^III^, MMA, and DMA followed by detection by inductively coupled plasma-mass spectrometry (ICP-MS) with dynamic reaction cell. Because As^III^ can oxidize to As^V^ during sample transport, storage, and preparation, we sum these two species to obtain total iAs (i.e., As^III^ + As^V^). The percentages of iAs, MMA and DMA in total arsenic were calculated by dividing the concentration of each species by the sum of iAs, MMA, and DMA. Data on arsenobetaine and aresenocholine, nontoxic organic arsenic from dietary sources, were not analyzed. Participants missing one of the four arsenic species were dropped from the analysis. *Generation of all urine arsenic species for HEALS and HEALS ancillary studies (FOX^14^, NIAT^32^, FACT^33^) were conducted at Columbia University’s trace elements lab, as described previously^34^, with the exception of ~1,800 HEALS urines samples which were more recently analyzed for arsenic species at the University of Alberta in the laboratory of Dr. Chris Le. Both labs use HPLC-ICP-MS to generate urine arsenic species measurements. Generation of all data on blood arsenic species used in this work has been described previously (for FOX^14^, NIAT^32^, FACT^33^). Bio-specimen measurements were averaged when data from multiple time points was available, as this increased power to detect known signals (AS3MT, FTCD)***.**

3. The pooled distributions of DMA%, MMA%, and iAs% in urine and blood from different studies are shown in Supplementary Figure 1. Please provide the distribution of arsenic species in different studies, so that the readers can better understand the differences of arsenic metabolism efficiency among different studies and different bio-samples.

**RESPONSE:** We now provide the following updated **Supplementary Figure 1** that shows the distribution of arsenic species by study, for both urine and blood. Overall, each arsenic species percentage has a very similar distribution across studies.

4. While the authors identified some associations between various SNPs and arsenic metabolism, and some of these SNPs are linked to arsenic-related skin lesions. Could the author include mediation analysis to further clarify the relationship between genetic variants, arsenic metabolism, and skin lesions?

**RESPONSE:** We have conducted mediation analyses for the 3 skin lesion-associated SNPs (rs4919690,rs191177668,rs6173583). This statement will be included in the results *GWAS of arsenic-induced skin lesions section:*

“*Mediation analyses were conducted for the 3 skin lesion-associated SNPs (rs4919690,rs191177668,rs6173583). We found that adjustment for DMA% substantially attenuates the association between these SNPs and skin lesion status (****Supplementary Table 4****). These results support the hypothesis that genetic variability in arsenic metabolism genes impacts arsenic-related toxicity risk, with AME acting as a mediato*r”

Supplementary Table 4. Mediation Analysis

| CHR | BP | SNP | Model | P | OR |
| --- | --- | --- | --- | --- | --- |
| 10 | 104616500 | **rs4919690 (AS3MT)** | unadjusted | 6.9x10^-10^ | 1.38 |
|  |  |  | DMA% adjusted | 0.06 | 1.15 |
| 10 | 104635687 | **rs191177668 (AS3MT)** | unadjusted | 1.7x10^-5^ | 2.32 |
|  |  |  | **DMA% adjusted** | 0.57 | 1.18 |
| 21 | 47572887 | **rs61735836 (FTCD)** | unadjusted | 4.1x10^-6^ | 1.36 |
|  |  |  | DMA% adjusted | 0.13 | 1.15 |

Reviewer #3: This is a well-conducted, clearly written study that aims to identify genetic loci that influence arsenic metabolism. The novel aspect of the study is the use of both blood and urine arsenic metabolites. The study population is a highly relevant one with high exposures and arsenic-related skin lesions. The methods and results are clearly presented.

My main comment to the authors is that I am not clear what the motivation for this analysis is, or what we have now learned from the study that would get us close to an end goal, which presumably, is to tailor interventions for reducing arsenic toxicity to groups based on their genetic profiles. These issues could be addressed with some minor revisions to the introduction and discussion.

For example, in the last paragraph of the introduction, the authors state, "Given the significant global health impact of arsenic exposure and the variability in AME among individuals, our objective is to identify novel genetic determinants of arsenic metabolism and toxicity phenotypes."

1. After reading the statement, my question was "Why?" -- how would this be a step toward an intervention. In the second paragraph of the discussion, "However, our new findings point to complexities in arsenic metabolism, distribution, transport and/or excretion that we do not yet fully understand." Yes, that's true, but why is that important? What implication could these have that might be important for the development of strategies that protect human health.

**RESPONSE:** To clarify the motivation for identifying genetic determinants of arsenic metabolism and toxicity and how this contributes to the development of interventions, we have expanded the introduction to explicitly state the translational significance of this work. Specifically, we emphasize how identifying genetic variants associated with arsenic metabolism can help us learn new aspects of AME biology and ultimately inform precision public health interventions in the last paragraph of the introduction and conclusion as specified below:

**Introduction:**

“In addition to AS3MT and FTCD, there are likely to be additional regions contributing to arsenic metabolism and toxicity risk (28). Given the significant global health impact of arsenic exposure and the variability in AME among individuals, our objective is to identify novel genetic determinants of arsenic metabolism and toxicity phenotypes. *Identifying such genetic determinants will implicate novel genes in AME, improving our understanding of biological processes underlying arsenic metabolism. Furthermore, knowledge of AME-associated variants could ultimately inform precision public health strategies focused on identifying highly susceptible groups who may benefit from targeted interventions, such as nutritional supplementation or enhanced exposure monitoring (*29, 30)*.* Here, we report the largest genome-wide association study (GWAS) of urine arsenic species conducted to date and the first GWAS of blood arsenic species to identify inherited genetic effects on arsenic metabolism and toxicity.”

Conclusion:

Future directions for this research include replication of the reported associations in other populations with arsenic exposure and evaluating our metabolism-related SNPs for association with other arsenic-related health outcomes. Additional research is also needed to characterize the molecular mechanisms by which the identified variants impact gene function, their relevant cellular contexts, and their roles in pathways involved in arsenic metabolism. *Importantly, identifying genetic variants associated with arsenic metabolism has the potential to inform precision public health strategies. By pinpointing genetically susceptible populations, these findings could support targeted interventions, such as nutritional supplementation or enhanced exposure monitoring, aimed at reducing arsenic-related health risks (*29, 30)*.*

To further strengthen this point, we have added a citation to our previous work:

Tamayo et al., *Returning personal genetic information on susceptibility to arsenic toxicity to research participants in Bangladesh* (2022), which demonstrates how GWAS findings on arsenic exposure can be translated into public health practice.

2. A very minor comment about terminology -- I am confused by the phrasing "GWAS of skin lesions," or "GWAS of urine species." It might be more precise to say a GWAS that focuses on detecting genetic variants that lead to differences in arsenic metabolites pattern in the urine (or something similar) at the first use, before going to the shorthand.

**Response:** To clarify, we conduct three types of GWAS in this paper (1) GWAS of arsenic species measured in urine, (2) GWAS of arsenic species measured in blood, and (3) GWAS of a arsenical skin lesions status (a case control analyses). We now ensure that we use very clear language when introducing and discussing each of those analyses and their respective results.
